# Changes in Alcohol Consumption and Associated Variables among Older Adults in Spain: A population-based cohort study

**DOI:** 10.1038/s41598-019-46591-0

**Published:** 2019-07-18

**Authors:** Hosanna Soler-Vila, Rosario Ortolá, Esther García-Esquinas, Luz Mª León-Muñoz, Fernando Rodríguez-Artalejo

**Affiliations:** 1 Department of Preventive Medicine and Public Health, School of Medicine, Universidad Autónoma de Madrid/IdiPAZ, C/Arzobispo Morcillo sn, Madrid, 28029 Spain; 2 CIBER of Epidemiology and Public Health (CIBERESP), Instituto de Salud Carlos III Melchor Fernández Almagro, 3-5, Madrid, 28029 Spain; 3 IMDEA-FOOD Institute, CEI UAM + CSIC, Madrid, 28029 Spain

**Keywords:** Epidemiology, Risk factors

## Abstract

We examined prospective changes in drinking patterns and their associations with socio-behavioral and health status variables in older adults in Spain using data from a prospective cohort of 2,505 individuals (53.3% women) representative of the non-institutionalized population aged >60 years in Spain. Alcohol consumption was assessed at baseline (2008–10) and at follow-up (2012) with a validated diet history. At risk drinking was defined as consuming >14 g of alcohol/day on average or any binge drinking in the last 30 days; lower amounts were considered light drinking. A total of 26.5% of study participants changed their intake during follow-up. Most participants reduced alcohol intake, but 23.3% of men and 8.9% of women went from light to at risk drinking during the study period. Low social connectivity at baseline was linked to at risk drinking for both sexes. However, the observed associations between changes in social connectivity, morbidity, BMI, or dietary habits and changes in drinking patterns differed by sex. We concluded that since about a quarter of older adults in Spain consume more alcohol than recommended, identifying socio-behavioral factors associated with this behavior is key for designing health campaigns targeting excessive alcohol consumption in this vulnerable population.

## Introduction

Alcohol consumption among older adults has been drawing increasing public health interest due to a rapidly growing elderly population and substantial evidence of a causal association between alcohol and disease burden^1–3^ even at substantially lower consumption levels than those previously deemed “safe”^4^. Due to physiological changes associated with ageing, older adults have a reduced tolerance to alcohol, may suffer ailments potentially aggravated by alcohol, and are likely to take prescriptions that can interact with alcohol. For these reasons, older adults are at increased risk of adverse effects from relatively modest levels of intake^5^. Although existing evidence points to a downward trend in alcohol consumption as people age^6,7^, some specifics are varying. Compared to previous cohorts, upcoming groups of older adults include a higher proportion of individuals reporting alcohol consumption over the recommended levels while the usual age-related decline observed has slowed down^8^. As a result, it is likely that the burden of disease from alcohol intake in older adults will increase in the future^3,9,10^. In fact, a significant number of older adults are consuming risky levels of alcohol for their age and prescription use^11^. Among U.S. Medicare recipients aged ≥65 years, 9% report consuming over 30 drinks per month or >3 drinks in one day^12^. In Spain, 5 to 8% of older adults diagnosed with hypertension, in treatment for diabetes or thrombosis, or taking sedatives report heavy drinking [≥40 g/day of alcohol in men and ≥24 g/day in women)^13^.

Several studies have assessed changes in alcohol intake over time, and their determinants, in young and middle-age adults [e.g.^14,15^], but far fewer studies have been conducted in older adults and, specifically, about the influence of social- and individual-level factors on alcohol intake [e.g.^7,10,16^]. After the age of 60 profound life transitions such as onset of chronic disease, overall functional deterioration, loss of spouse and other family members, retirement, and weakening social and familiar ties are more likely to come about. These transitions and changes in social networks both define the social context of drinking and could influence consumption patterns as alcohol is often used as a stress-buffering mechanism^7,17–19^. A deeper understanding of these issues could guide interventions to prevent excessive alcohol intake in older adults.

Thus, the aim of this paper is threefold. First, we examine how drinking patterns vary in the Spanish older adult population between two time-points in a 3-year period. Secondly, we explore how baseline socio-behavioral and health status variables are prospectively associated with 3-year changes in alcohol consumption. Finally, we identify how changes in these same variables throughout the 3 years are associated with concurrent changes in alcohol intake. Given the gendered social context surrounding alcohol and the unequal distribution of both consumption and of related disease burden by sex^2,7,20^, these objectives are addressed for men and women separately.

## Methods

### Study design and participants

The study design is described in detail elsewhere^21,22^. Briefly, data come from the Seniors-ENRICA cohort, a longitudinal study including 2,614 individuals selected in 2008–10 through stratified cluster random sampling of the non-institutionalized population of Spain aged 60 years and older. First, the sample was stratified by province and size of municipality. Second, clusters were selected randomly in two stages: municipalities and census sections. Finally, the households within each section were selected by random telephone dialling based on the directory of telephone landlines. Subjects within the households were selected proportionally to the sex and age distribution of the Spanish population.

At baseline, we collected comprehensive information on socio-demographic variables, health behaviors, health status indicators and morbidity through computer-assisted telephone interviews and structured questionnaires. Additionally, trained staff conducted two home visits to record a complete diet and alcohol history, to perform a physical examination, and to collect blood and urine samples.

About 3 years later [February through December 2012), 2,519 surviving participants agreed to participate in a follow-up through telephone and at-home interviews for data updates. All study participants provided written informed consent. The study protocol was approved by the Clinical Research Ethics Committee of the University Hospital *La Paz* in Madrid^21,22^. This research has been performed in accordance with the ethical standards described in the 1964 Declaration of Helsinki and its later amendments.

### Study variables

#### Alcohol consumption

Both at baseline and follow-up, participants were administered a validated diet history, based on the one used in the EPIC-cohort study^23–25^. As part of this diet history, we collected detailed information on habitual average alcohol consumption in the preceding 12 months including binge drinking in the last 30 days.

We classified participants of either gender as “at risk drinkers” if they reported consuming more than one U.S. standard drink (>14 g of alcohol) per day on average^5,26^ or any binge drinking in the last 30 days (≥80 g for men and ≥60 g for women of alcohol in one session)^27^. Individuals reporting lower consumption, labeled here as light drinkers, correspond to drinkers with the lowest risk of all-cause mortality according to Wood and colleagues^4^. Non-drinkers included life-long abstainers and very occasional drinkers (i.e., individuals who reported 0 g/day of alcohol intake in the last year but self-described as drinkers). We classified as ex drinkers those individuals who expressed having quit alcohol consumption and reported 0 g/day of alcohol intake in the past 12 months. We differentiated between ex drinkers and non-drinkers because the former may have quit alcohol due to previous health issues (the sick quitter effect) or as a conscious choice for a healthier lifestyle^4^.

We defined three patterns of drinking change over time for which we had large enough sample sizes: Light drinker at baseline but at risk drinker at follow-up (Light-to-At Risk), light drinker at baseline but ex drinker at follow-up (Light-to-Ex drinker), and at risk drinker at baseline but light drinker at follow-up (At Risk-to-Light). These three categories were compared to those participants who maintained their baseline pattern throughout follow-up. That is, if they were light drinkers at baseline, they also reported light drinking at follow-up and if they were at risk drinkers at baseline, they remained at risk drinkers at follow-up.

#### Socio-behavioral and health status variables

We included baseline and follow-up variables previously associated with changes in alcohol consumption, including gender, age, educational level, tobacco smoking, marital and employment status, leisure time exercise (expressed in metabolic equivalents-METS), sedentary behavior (time spent watching television or reading per week), obesity, adherence to the Mediterranean diet as per the MEDAS index, physical (PCS) and mental (MCS) health-related quality of life based on the SF-12 questionnaire, social support or connectivity (living alone, eating meals alone) [e.g.^7,15,19,22,28,29^] and the number of physician-diagnoses of the following diseases or conditions: pneumonia, asthma, chronic bronchitis, heart attack, stroke, cardiac failure, osteoarthritis, rheumatoid arthritis, hip fracture, gallbladder stones, cirrhosis of the liver, urinary tract infection, depression requiring treatment, Parkinson’s disease, dementia, gum disease, and/or cancer. The presence of any of these conditions may have influenced alcohol consumption^30^ or time spent socializing around alcohol. Participants also reported whether a health care provider had diagnosed them with hypertension, diabetes, and/or high cholesterol levels. We used standard procedures to measure weight and height^31^ from which we calculated Body Mass Index (BMI) as weight in kg divided by squared height in m.

We calculated change variables using baseline and follow-up values. In the case of continuous variables, we selected cut-points reflecting clinically significant thresholds or the median value at baseline. These values are specified in the corresponding tables.

### Statistical analysis

At the bivariate level, we calculated the percentage of participants falling into each of the four consumption patterns at baseline and follow-up. Because aggregate data tend to mask the degree of variation in alcohol consumption over time, we looked at the path participants followed between drinking categories from baseline to follow-up. Given the substantial differences in drinking patterns by gender, all analyses were performed for men and women separately.

At the multivariate level, the dependent variable consisted of each of the two change patterns described above for light drinkers (Light-to-At Risk vs. Continuing Light; Light-to-Ex drinker vs. Continuing Light) plus only one change pattern regarding at risk drinkers (At Risk-to-Light vs. Continuing At Risk) for lack of enough individuals reporting At Risk drinking at baseline and then reporting having quit drinking at follow-up. Thus, we examine a total of three change drinking patterns. In a first model, we summarized the associations of socio-behavioral and health status, and social support variables at baseline with these three changes in drinking patterns using multivariate logistic regression models. These analyses yielded odds ratios (OR) and their corresponding 95% confidence interval (CI) fully adjusted for the other independent variables of interest in addition to age and baseline alcohol intake. As a next step, we incorporated a set of independent variables capturing changes in socio-behavioral and health variables from baseline to follow-up to the first model. This second model allowed us to estimate relationships between these changes and changes in drinking patterns while controlling for the effect of starting points, i.e., baseline values.

All variables were modeled as categorical using dummy terms, except for the variable capturing the body mass index and the number of morbidities which were kept continuous. Statistical significance was set at two-sided p < 0.05. We used STATA version 11.1 (StataCorp. LP, College Station, 2010) to perform the analyses.

## Results

### Description of the sample

Of the 2,614 participants from the baseline study, 95 died before the end of follow-up. We also excluded 14 people for lack of data on alcohol consumption, MEDAS, BMI, employment status, or the SF-12. Thus, main analyses were conducted on 2,505 individuals (1,171 men and 1,334 women; 1,546 aged 60–69 years and 959 aged ≥70 years). Almost a quarter had completed secondary level education and one fifth reported university-level degrees. At baseline, 71.5% were married and 18.5% were widowed. Two-thirds were retired (66.3%) and almost 12% were still employed (Table 1).

**Table 1:** Patterns of alcohol consumption in 2008–10 (T1) and 2012 (T2) among individuals 60 years and older in Spain, by socio-demographic characteristics.

Characteristics at T1	Total n (%)	Non-Drinker n (%)		Ex Drinker n (%)		Light Drinker n (%)		At Risk Drinker n (%)	
		T1	T2	T1	T2	T1	T2	T1	T2
	2505 (100)	757 (30.2)	590 (23.6)	208 (8.3)	266 (10.6)	900 (35.9)	1050 (41.9)	640 (25.5)	599 (23.9)
**Age (yrs)**									
Mean age	68.7	69.5	72.9	71.3	73.8	68.3	71.5	67.5	71.0
**Gender, n (%)**									
Men	1171 (46.8)	126 (16.6)	98 (16.6)	104 (0.5)	118 (44.4)	438 (48.7)	481 (45.8)	503 (78.6)	474 (79.1)
Women	1334 (53.3)	631 (83.4)	492 (83.4)	104 (0.5)	148 (55.6)	462 (51.3)	569 (54.2)	137 (21.4)	125 (20.9)
**Education, n (%)**									
University	527 (21.0)	110 (14.5)	83 (14.1)	41 (19.7)	46 (17.3)	207 (23.0)	229 (21.8)	169 (26.4)	169 (28.2)
Secondary	611 (24.4)	139 (18.4)	105 (17.8)	41 (19.7)	53 (19.9)	237 (26.3)	283 (27.0)	194 (30.3)	170 (28.4)
Primary	1367 (54.6)	508 (67.1)	402 (68.1)	126 (60.6)	167 (62.8)	456 (50.7)	538 (51.2)	277 (43.3)	260 (43.4)
**Marital Status, n (%)**									
Married	1792 (71.5)	468 (61.8)	365 (61.9)	148 (71.2)	190 (71.4)	660 (73.3)	766 (73.0)	516 (80.6)	471 (78.6)
Not Married	250 (10.0)	72 (9.5)	55 (9.3)	16 (7.7)	16 (6.0)	96 (10.7)	111 (10.6)	66 (10.3)	68 (11.4)
Widowed	463 (18.5)	217 (28.7)	170 (28.8)	44 (21.2)	60 (22.6)	144 (16.0)	173 (16.5)	58 (9.1)	60 (10.0)
**Employment, n (%)**									
Retired	1660 (66.3)	418 (55.2)	334 (56.6)	144 (69.2)	173 (65.0)	598 (66.4)	678 (64.6)	500 (78.1)	475 (79.3)
Employed	292 (11.7)	64 (8.5)	46 (7.8)	21 (10.1)	19 (7.1)	114 (12.7)	144 (13.7)	93 (14.5)	83 (13.9)
Unemployed	553 (22.1)	275 (36.3)	210 (35.6)	43 (20.7)	74 (27.8)	188 (20.9)	228 (21.7)	47 (7.3)	41 (6.8)
**Alcohol Intake (g/day)**									
Mean alcohol intake		0	0	0	0	4.6	5.0	34.0	28.5

### Frequency of main drinking patterns at 2008–10 (T1) and 2012 (T2) by socio-demographic variables

We observed substantial differences in drinking patterns between men and women; for example, women made up a much higher percentage of non-drinkers than men at T1 (83.4 vs. 16.6%), whereas the reverse was true for at risk drinking (21.4 vs. 78.6%). The frequency of light drinkers increased moderately from T1 to T2 whereas the number of non-drinkers decreased to a similar extent. The numbers of ex drinkers and at risk drinkers showed little to no change. The average amount of alcohol consumption increased minimally among light drinkers (0.4 g/day) whereas consumption decreased by 5.5 g/day among at risk drinkers (Table 1).

### Individual changes in drinking patterns from T1 to T2

In Tables 2 and 3 we compared drinking patterns reported at T1 (leftmost column) with T2 patterns (top row). Zeros in tables reflect non-viable pattern changes, namely, from any drinking category to non-drinking and from non-drinker to ex drinker. Among men, the ex-drinking and light drinking categories experienced moderate gains (+13.5% and +9.8%, respectively) by drawing from loses in the non-drinking and at risk drinking categories (−22.2% and −5.8%, respectively). In total, 28.6% of men changed the drinking pattern during the 3-year follow-up. Whereas 22.3% (16.7% + 5.6%) of non-drinkers and 28.9% (23.1% + 5.8%) of ex drinkers at T1 reported measurable alcohol consumption (i.e., light or at risk) at T2, 40.5% of all men reported at risk drinking at T2 down from 43.0% at baseline. Among light drinkers at T1, 23.3% became at risk drinkers at T2 (Table 2).

**Table 2:** Changes in pattern of alcohol consumption between 2008-2010 (T1) and 2012 (T2) among **men** 60 years and older in Spain.

Men	Alcohol consumption T2								Total T1	
Alcohol consumption T1	Non-drinker		Ex-drinker		Light drinker		At Risk drinker			
	n	%	n	%	n	%	n	%	n	(column %)
Non-drinker, n, row %	98	77.8	0	0.0	21	16.7	7	5.6	126	10.8
Ex drinker, n, row %	0	0.0	74	71.2	24	23.1	6	5.8	104	8.9
Light drinker, n, row %	0	0.0	31	7.1	305	69.6	102	23.3	438	37.4
At Risk drinker, n, row %	0	0.0	13	2.6	131	26.0	359	71.4	503	43.0
**Total T2, n, row %**	98	8.4	118	10.1	481	41.1	474	40.5	**1171**	**100.0**
**Change, n**,^**a**^ **%**^**b**^	−28	−22.2	+14	+13.5	+43	+9.8	−29	−5.8		

^a^Total T2-Total T1; ^b^(T2-T1)/T1.

**Table 3:** Changes in pattern of alcohol consumption between 2008–2010 (T1) and 2012 (T2) among **women** 60 years and older in Spain.

Women	Alcohol consumption T2								Total T1	
Alcohol consumption T1	Non-drinker		Ex-drinker		Light drinker		At Risk drinker			
	n	%	n	%	n	%	n	%	n	(column %)
Non-drinker, n, row %	492	78.0	0	0.0	132	20.9	7	1.1	631	47.3
Ex drinker, n, row %	0	0.0	80	76.9	21	20.2	3	2.9	104	7.8
Light drinker, n, row %	0	0.0	61	13.2	360	77.9	41	8.9	462	34.6
At Risk drinker, n, row %	0	0.0	7	5.1	56	40.9	74	54.0	137	10.3
**Total T2, n, row %**	492	36.9	148	11.1	569	42.7	125	9.4	**1334**	**100.0**
**Change, n**,^**a**^ **%**^**b**^	−139	−22.0	+44	+42.3	+107	+23.2	12	−8.8		

^a^Total T2-Total T1; ^b^(T2-T1)/T1.

Among women, the group that gained the most individuals during the study period was the ex drinkers (+42.3%), followed by light drinkers (+23.2%). These two categories absorbed those non-drinkers and at risk drinkers who switched patterns (−22.0% and −8.8%, respectively). Overall 24.5% changed drinking patterns over the study’s follow-up. Whereas 22.0% of non-drinkers (20.9% + 1.1%) and 23.1% of ex drinkers (20.2% + 2.9%) at T1 reported measurable alcohol consumption at T2, only 9.4% of all women reported at risk drinking at T2, slightly lower than the 10.3% reported at baseline. Among light drinkers at T1, 8.9% became at risk drinkers at T2 (Table 3).

### How are socio-demographic and health-related characteristics at baseline associated with changes in drinking patterns in the following 3 years?

Among male light drinkers at baseline, those living alone were substantially more likely to reach at risk drinking by follow-up than those living with someone (OR 4.72; 95%CI 1.48–14.99). Also, each additional morbidity at baseline was associated with a greater likelihood of quitting alcohol between baseline and follow-up (OR 1.41; 1.01–1.99) (Table 4).

**Table 4:** Odds ratios (95% confidence interval) for the association of socio-behavioral and health status variables with changes in drinking from baseline (T1) to follow-up (T2) among **men** 60 and older in Spain (N = 928).

T1 VARIABLES	Light-to-At risk vs. continuing Light (102 vs. 305)	Light-to-Ex drinker vs. continuing Light (31 vs. 305)	At risk-to-Light vs. continuing At risk (131 vs. 359)
	OR^a^ (95% CI)	OR^a^ (95% CI)	OR^a^ (95% CI)
**Socio-demographic**			
70 and older vs. 60–69	0.97 (0.57–1.64)	1.65 (0.69–3.98)	0.71 (0.43–1.17)
Retired vs. other	1.19 (0.58–2.43)	0.42 (0.15–1.14)	1.14 (0.64–2.05)
University studies vs. secondary or less	0.87 (0.49–1.54)	0.86 (0.32–2.34)	0.69 (0.42–1.14)
Not married vs married	1.05 (0.42–2.65)	0.38 (0.05–3.07)	0.60 (0.23–1.57)
**Lifestyle**			
Current smoker vs. not	1.24 (0.62–2.49)	1.67 (0.59–4.72)	0.67 (0.39–1.16)
≥9 MEDAS score vs. <9	0.86 (0.50–1.47)	0.82 (0.33–2.07)	0.80 (0.51–1.28)
≥Median LTPA vs. Median (METS)	1.22 (0.70–2.10)	1.47 (0.59–3.64)	0.76 (0.48–1.20)
≥Median reading time vs. <Median	1.14 (0.70–1.87)	0.46 (0.20–1.07)	0.97 (0.62–1.51)
≥Median TV time vs. <Median	1.20 (0.70–2.06)	1.18 (0.45–3.12)	1.29 (0.82–2.04)
**Health Status**			
Body Mass Index	1.01 (0.94–1.07)	0.94 (0.84–1.05)	1.06 (1.00–1.12)
Number of morbidities^b^	0.82 (0.61–1.10)	**1.41 (1.01–1.99)**	0.81 (0.60–1.09)
≥Median PCS of the SF-12 vs. <Median	0.77 (0.45–1.31)	0.80 (0.32–2.03)	1.41 (0.87–2.28)
≥Median MCS of the SF-12 vs. <Median	1.04 (0.64–1.69)	0.63 (0.28–1.43)	1.06 (0.69–1.64)
**Social Support**			
Eat meals alone (always vs. not always)	0.51 (0.24–1.10)	0.80 (0.23–2.78)	1.13 (0.54–2.38)
Living situation (alone vs. not)	**4.72 (1.48–14.99)**	5.00 (0.50–49.65)	0.77 (0.20–2.99)

OR, Odds ratio; CI, Confidence Interval; MEDAS, Mediterranean Diet Adherence Screener; LTPA, Leisure Time Physical Activity; METS, Metabolic Equivalent of Task; TV, television; PCS, Physical Component Summary; MCS Mental Component Summary; SF-12, the 12-Item Short Form.

^a^Multivariate ORs are adjusted for all other variables in the table plus alcohol consumption (g/day) at baseline. Statistically significant ORs are shown in **bold**.

^b^Morbidities included: Pneumonia, asthma, chronic bronchitis, heart attack, stroke, cardiac failure, osteoarthritis, rheumatoid arthritis, hip fracture, gallbladder stones, cirrhosis of the liver, urinary tract infection, depression requiring treatment, Parkinson’s disease, dementia, gum disease, cancer.

As regards to female light drinkers, those who had all their meals alone more frequently adopted at risk intake at follow-up than those having company at least some times (OR 2.91; 1.17–7.29). Finally, of those presenting at risk drinking at baseline, older women and those spending more time reading were more likely to reduce their intake from at risk to light levels (OR 2.63; 1.05–6.61 and 2.60; 1.08–6.27, respectively) (Table 5).

**Table 5:** Odds ratios (95% confidence interval) for the association of socio-behavioral and health status variables with changes in drinking categories from baseline (T1) to follow-up (T2) among **women** 60 and older in Spain (N = 592).

T1 VARIABLES	Light-to-At risk vs. continuing Light (41 vs. 360)	Light-to-Ex drinker vs. continuing Light (61 vs. 360)	At risk-to-Light vs. continuing At risk (56 vs. 74)
	OR^a^ (95% CI)	OR^a^ (95% CI)	OR^a^ (95% CI)
**Socio-demographic**			
70 and older vs. 60–69	0.58 (0.24–1.39)	1.35 (0.73–2.51)	2.63 (1.05–6.61)
Retired vs. other	1.70 (0.80–3.64)	0.84 (0.45–1.59)	0.52 (0.22–1.23)
University studies vs. secondary or less	1.60 (0.73–3.50)	0.82 (0.35–1.95)	0.52 (0.17–1.61)
Not married vs married	0.84 (0.31–2.28)	0.38 (0.14–1.03)	0.58 (0.19–1.73)
**Lifestyle**			
Current smoker vs. not	2.07 (0.72–5.92)	0.53 (0.12–2.40)	0.34 (0.07–1.53)
≥9 MEDAS score vs. <9	0.62 (0.24–1.62)	1.22 (0.61–2.44)	0.98 (0.38–2.50)
≥Median LTPA vs. <Median (METS)	0.58 (0.29–1.16)	0.62 (0.35–1.10)	0.52 (0.23–1.19)
≥Median reading time vs. <Median	0.98 (0.48–1.97)	0.78 (0.43–1.42)	**2.60 (1.08–6.27)**
≥Median TV time vs. <Median	0.58 (0.28–1.20)	0.91 (0.47–1.75)	1.01 (0.37–2.76)
**Health Status**			
Body Mass Index	0.97 (0.89–1.06)	1.02 (0.95–1.09)	0.96 (0.87–1.06)
Number of morbidities^b^	1.07 (0.77–1.48)	1.10 (0.84–1.43)	1.15 (0.80–1.65)
≥Median PCS of the SF-12 vs. <Median	1.18 (0.56–2.46)	0.62 (0.32–1.18)	1.42 (0.60–3.35)
≥Median MCS of the SF-12 vs. <Median	1.58 (0.78–3.20)	0.69 (0.37–1.31)	1.53 (0.66–3.56)
**Social Support**			
Eat meals alone (always vs. not always)	**2.91 (1.17–7.29)**	1.70 (0.73–3.99)	1.51 (0.40–5.62)
Living situation (alone vs. not)	0.81 (0.26–2.56)	1.97 (0.63–6.14)	1.70 (0.40–7.15)

OR, Odds ratio; CI, Confidence Interval; MEDAS, Mediterranean Diet Adherence Screener; LTPA, Leisure Time Physical Activity; METS, Metabolic Equivalent of Task; TV, television; PCS, Physical Component Summary; MCS Mental Component Summary; SF-12, the 12-Item Short Form.

^a^Multivariate ORs are adjusted for all other variables in the table plus alcohol consumption (g/day) at baseline. Statistically significant ORs are shown in **bold**.

^b^Morbidities included: Pneumonia, asthma, chronic bronchitis, heart attack, stroke, cardiac failure, osteoarthritis, rheumatoid arthritis, hip fracture, gallbladder stones, cirrhosis of the liver, urinary tract infection, depression requiring treatment, Parkinson’s disease, dementia, gum disease, cancer.

### How are changes in socio-demographic and health-related characteristics between baseline and follow-up associated with changes in drinking patterns during the same period?

Male light drinkers. Among male light drinkers, those who abandoned the Mediterranean diet between baseline and follow-up more than double their likelihood of at risk consumption 3 years later (OR for Light-to-At Risk: 2.29; 1.16–4.50). In contrast, a decrease in time spent reading showed the opposite relationship (OR for Light-to-At Risk: 0.40; 0.17–0.92). Also, each additional morbidity at baseline was inversely associated with at risk consumption (Light-to-At Risk OR: 0.54; 0.35–0.83), and a reduction in number of morbidities during follow-up was linked to more frequent adoption of an at risk pattern (Light-to-At Risk OR: 3.75; 1.60–8.79) independently of how many diagnoses were reported at baseline. However, a decline in the physical component of the SF-12 was positively associated with increased consumption (OR Light-to-At Risk: 1.92; 1.03–3.59) (Table 6).

**Table 6:** Odds ratios (95% confidence interval), for the association of changes in socio-behavioral and health status variables with changes in drinking categories from baseline (T1) to follow-up (T2) among **men** 60 and older in Spain (N = 928).

	Light-to-At risk vs. continuing Light (102 vs. 305)	Light-to-Ex drinker vs. continuing Light (31 vs. 305)	At risk-to-Light vs. continuing At risk (131 vs. 359)
	OR^a^ (95% CI)	OR^a^ (95% CI)	OR^a^ (95% CI)
**Socio-demographic, T1**			
T1 70 and older vs. 60–69	0.93 (0.53–1.63)	1.08 (0.38–3.12)	0.81 (0.47–1.37)
T1 University studies vs. secondary or less	0.87 (0.48–1.59)	0.85 (0.27–2.67)	0.68 (0.40–1.15)
**Socioeconomic, T1 and T1-T2 change**			
T1 Retired vs. other	2.43 (0.60–9.78)	0.51 (0.08–3.47)	1.09 (0.40–2.96)
Entered retirement vs. other^b^	2.32 (0.50–10.89)	1.85 (0.22–15.45)	1.26 (0.40–3.94)
T1 Not married vs married	1.91 (0.92–3.96)	0.37 (0.06–2.45)	0.61 (0.27–1.38)
Lost spouse vs. other^c^	1.50 (0.30–7.62)	0.51 (0.03–9.29)	0.79 (0.15–4.21)
**Lifestyle, T1 and T1-T2 change (vs. no change)**			
T1 Current smoker vs. not	1.47 (0.58–3.71)	3.44 (0.75–15.81)	**0.42 (0.20–0.87)**
Quitted smoking^d^	0.59 (0.14–2.45)	0.20 (0.01–3.53)	**3.20 (1.09–9.38)**
T1 LTPA ≥ Median vs. <Median (METS)	1.15 (0.62–2.11)	1.08 (0.33–3.52)	0.89 (0.53–1.50)
Decreased LTPA^e^	1.53 (0.75–3.10)	2.32 (0.60–9.01)	0.85 (0.45–1.61)
Increased LTPA^e^	1.45 (0.68–3.09)	1.17 (0.31–4.41)	1.39 (0.75–2.57)
T1 Reading time ≥ Median vs. <Median	1.79 (0.91–3.48)	0.37 (0.12–1.12)	0.74 (0.43–1.28)
Decrease in reading time^e^	**0.40 (0.17–0.92)**	4.51 (0.75–27.01)	1.83 (0.84–4.02)
Increase in reading time^e^	0.93 (0.25–3.42)	4.71 (0.52–42.59)	0.88 (0.22–3.42)
T1 time watching TV ≥ Median vs. <Median	0.86 (0.47–1.58)	1.57 (0.48–5.12)	1.83 (1.07–3.12)
Decrease in TV watching time^e^	1.04 (0.54–2.00)	0.83 (0.18–3.76)	1.59 (0.84–2.98)
Increase in TV watching time^e^	0.53 (0.28–1.01)	1.96 (0.57–6.75)	**2.00 (1.10–3.65)**
**T1-T2 Change in adherence to Mediterranean Diet** ^**f,g**^			
Lost adherence	**2.29 (1.16–4.50)**	0.18 (0.21–1.62)	0.74 (0.39–1.40)
Achieved adherence	0.94 (0.41–2.16)	2.50 (0.68–9.21)	1.89 (0.99–3.61)
**Health Status, T1 and T1-T2 change (vs. no change)**			
T1 Body Mass Index (BMI)	1.03 (0.96–1.10)	**0.87 (0.77–0.99)**	**1.08 (1.01–1.15)**
Reduction in BMI^h^	1.12 (0.55–2.29)	**9.71 (2.95–31.96)**	1.40 (0.77–2.55)
Increase in BMI^h^	1.41 (0.52–3.78)	0.72 (0.07–7.85)	**2.28 (1.01–5.16)**
T1 Number of morbidities^i^	**0.54 (0.35–0.83)**	**1.77 (1.04–3.00)**	0.73 (0.49–1.07)
Reduction in number of morbidities	**3.75 (1.60–8.79)**	2.12 (0.47–9.68)	1.41 (0.65–3.05)
Increase in number of morbidities	0.86 (0.47–1.58)	**3.25 (1.05–10.05)**	1.11 (0.66–1.85)
T1 PCS of the SF-12≥ Median vs. <Median	0.89 (0.48–1.66)	0.76 (0.26–2.21)	1.30 (0.74–2.27)
Decline in PCS score of the SF-12^j^	**1.92 (1.03–3.59)**	1.30 (0.40–4.25)	0.81 (0.47–1.39)
Improvement in PCS score of the SF-12^j^	1.31 (0.66–2.61)	1.13 (0.31–4.14)	0.70 (0.37–1.30)
T1 MCS of the SF-12≥ Median vs. <Median	1.02 (0.58–1.80)	0.51 (0.19–1.40)	0.88 (0.52–1.49)
Decline in MCS score of the SF-12^j^	1.07 (0.56–2.03)	0.36 (0.10–1.34)	1.07 (0.60–1.91)
Improvement in MCS score of the SF-12^j^	0.64 (0.33–1.24)	0.50 (0.15–1.64)	0.83 (0.45–1.52)
**T1-T2 Change in Social Support (vs. no change)**			
Usual meal consumption^f^			
Meals alone to meals with company	0.71 (0.30–1.69)	1.45 (0.35–6.01)	1.24 (0.49–3.11)
Meals with company to meals alone	2.18 (0.78–6.10)	0.02 (0.00–10.27)	**3.03 (1.12–8.20)**
Living situation^f^			
Living alone to living with someone	0.40 (0.04–4.55)	—	1.90 (0.15–23.68)
Living with someone to living alone	0.47 (0.11–1.95)	—	0.38 (0.07–2.09)

OR, Odds ratio; CI, Confidence Interval; MEDAS, Mediterranean Diet Adherence Screener; LTPA, Leisure Time Physical Activity; METS, Metabolic Equivalent of Task, TV, television; PCS, Physical Component Summary; MCS Mental Component Summary; SF-12, the 12-Item Short Form. “—” cell size too small for analysis.

^a^Multivariate ORs are adjusted for all other variables shown in the table, both baseline and change variables, plus alcohol consumption (g/day) at baseline. Statistically significant ORs are shown in **bold**. ^b^“Other” includes other variations in employment status as well as “no change”; ^c^“Lost spouse” due to divorce or death vs. “Other” (got married or no change in status); ^d^The reference category includes participants who reported “no change” and a small number who started smoking; ^e^Change was defined as a decrease or increase of at least 15% of the baseline value; ^f^Baseline variable removed from the model due to collinearity with the change variable; ^g^Adherence to the Mediterranean diet is defined by a MEDAS score ≥9; ^h^Change in BMI was defined as a reduction or increase of at least 5% of the baseline value; ^i^Change was defined as a reduction or increase of number of morbidities by 1 vs baseline. Morbidities included: Pneumonia, asthma, chronic bronchitis, heart attack, stroke, cardiac failure, osteoarthritis, rheumatoid arthritis, hip fracture, gallbladder stones, cirrhosis of the liver, urinary tract infection, depression requiring treatment, Parkinson’s disease, dementia, gum disease, and cancer; ^j^Change was defined as at least a 3-point decline or improvement in score compared to baseline score.

A reduction in BMI was more likely among those quitting alcohol during follow-up (OR for Light-to-Ex drinker: 9.71; 2.95–31.96) regardless of baseline BMI, which was itself inversely associated with quitting consumption (OR for Light-to-Ex drinker: 0.87; 0.77–0.99). And again, an increase in morbidities during follow-up showed an association with tapering intake during the same time period (OR for Light-to-Ex drinker: 3.25; 1.05–10.05) net of baseline comorbidity, which remained directly associated with quitting drinking (OR for Light-to-Ex drinker: 1.77; 1.04–3.00) (Table 6).

#### Male at risk drinkers

Men reporting at risk consumption and who reported smoking at baseline were more likely than non- and ex-smokers to continue at risk alcohol consumption rather than switching to light drinking (OR for At Risk-to-Light: 0.42; 0.20–0.87); further, those who quitted tobacco during follow-up were substantially more likely than their counterparts to also quit at risk drinking in favor of light drinking (OR for At Risk-to-Light: 3.20; 1.09–9.38). Baseline BMI was positively associated to improving consumption pattern from at risk to light drinking (OR: 1.08; 1.01–1.15); and, independently of baseline BMI, men who gained weight during follow-up were also more likely to report reducing consumption (OR for At Risk-to-Light: 2.28; 1.01–5.16). Increasing time watching TV during those 3 years was associated to a healthier drinking profile (OR for At Risk-to-Light: 2.00; 1.10–3.65). Lastly, compared to those who did not change their meal habits, individuals who changed from eating in the company of others (at least sometimes) at baseline to always eating alone at follow-up were more likely to reduce their drinking (OR for At Risk-to-Light: 3.03; 1.12–8.20) (Table 6).

#### Female light drinkers

Among women reporting light drinking at baseline, those who reduced their company during meals to always eating alone at follow-up were more likely to quit drinking (OR for Light-to-Ex drinker: 4.13; 1.34–12.74) (Table 7).

**Table 7:** Odds ratios (95% confidence interval), for the association of changes in socio-behavioral and health status variables with changes in drinking categories from baseline (T1) to follow-up (T2) among **women** 60 and older in Spain (N = 592).

	Light-to-At risk vs. continuing Light (41 vs. 360)	Light-to-Ex drinker vs. continuing Light (61 vs. 360)	At risk-to-Light vs. continuing At risk (56 vs. 74)
	OR^a^ (95% CI)	OR^a^ (95% CI)	OR^a^ (95% CI)
**Socio-demographic, T1**			
T1 70 and older vs. 60–69	0.55 (0.22–1.38)	1.26 (0.64–2.48)	**4.64 (1.51–14.26)**
T1 University studies vs. secondary or less	1.58 (0.68–3.68)	0.78 (0.30–2.00)	0.49 (0.14–1.74)
**Socioeconomic, T1 and T1-T2 change**			
T1 Retired vs. other	1.49 (0.53–4.14)	0.63 (0.28–1.40)	**0.21 (0.05–0.95)**
Entered retirement vs. other^b^	0.72 (0.22–2.40)	0.68 (0.29–1.63)	0.79 (0.15–4.08)
T1 Not married vs married	1.43 (0.63–3.26)	0.70 (0.34–1.45)	0.85 (0.26–2.77)
Lost spouse vs. other^c^	2.41 (0.47–12.43)	0.53 (0.08–3.57)	0.26 (0.01–4.47)
**Lifestyle, T1 and T1-T2 change (vs. no change)**			
T1 Current smoker vs. not	1.84 (0.42–8.14)	0.22 (0.02–2.06)	0.47 (0.06–3.96)
Quitted smoking^d^	0.82 (0.10–6.80)	3.78 (0.15–96.96)	1.34 (0.05–36.86)
T1 LTPA ≥Median vs. <Median (METS)	0.69 (0.31–1.54)	0.75 (0.38–1.45)	0.56 (0.20–1.58)
Decreased LTPA^e^	1.96 (0.60–6.39)	1.71 (0.68–4.29)	0.58 (0.14–2.43)
Increased LTPA^e^	1.80 (0.57–5.74)	1.65 (0.66–4.16)	1.43 (0.35–5.95)
T1 Reading time ≥Median vs. <Median	1.15 (0.54–2.43)	1.05 (0.50–2.18)	3.54 (0.89–14.10)
Decrease in reading time^e^	—	0.80 (0.35–1.85)	0.95 (0.22–4.07)
Increase in reading time^e^	—	1.84 (0.42–8.08)	0.95 (0.07–13.62)
T1 time watching TV ≥ Median vs. <Median	0.78 (0.33–1.88)	1.11 (0.53–2.33)	1.89 (0.51–7.03)
Decrease in TV watching time^e^	1.00 (0.32–3.13)	0.70 (0.29–1.72)	0.49 (0.12–1.97)
Increase in TV watching time^e^	1.88 (0.71–5.01)	1.48 (0.69–3.17)	1.33 (0.35–5.10)
**T1-T2 Change in adherence to Mediterranean Diet** ^**f,g**^			
Lost adherence	2.07 (0.81–5.33)	1.70 (0.70–4.07)	**4.28 (1.06–17.31)**
Achieved adherence	0.18 (0.02–1.48)	1.66 (0.66–4.16)	4.50 (0.96–21.05)
**Health Status, T1 and T1-T2 change (vs. no change)**			
T1 Body Mass Index (BMI)	0.97 (0.88–1.07)	1.03 (0.95–1.11)	0.95 (0.84–1.07)
Reduction in BMI^h^	1.39 (0.48–3.98)	1.97 (0.88–4.41)	2.13 (0.61–7.43)
Increase in BMI^h^	1.46 (0.50–4.28)	0.84 (0.30–2.34)	0.70 (0.07–6.95)
T1 Number of morbidities^i^	1.02 (0.66–1.59)	1.17 (0.86–1.61)	1.45 (0.88–2.37)
Reduction in number of morbidities	1.27 (0.45–3.64)	1.16 (0.47–2.90)	0.81 (0.21–3.03)
Increase in number of morbidities	0.67 (0.27–1.67)	2.06 (0.98–4.34)	1.39 (0.38–5.06)
T1 PCS of the SF-12 ≥Median vs. <Median	1.27 (0.54–2.97)	0.63 (0.31–1.30)	1.94 (0.65–5.77)
Decline in PCS score of the SF-12^j^	0.56 (0.21–1.48)	0.67 (0.31–1.42)	0.36 (0.10–1.29)
Improvement in PCS score of the SF-12^j^	0.84 (0.31–2.24)	0.53 (0.23–1.23)	0.98 (0.28–3.42)
T1 MCS of the SF-12 ≥Median vs. <Median	1.29 (0.56–2.99)	0.67 (0.33–1.37)	2.42 (0.75–7.88)
Decline in MCS score of the SF-12^j^	1.54 (0.57–4.14)	0.97 (0.42–2.23)	0.72 (0.20–2.63)
Improvement in MCS score of the SF-12^j^	1.47 (0.50–4.26)	1.26 (0.55–2.89)	1.72 (0.38–7.83)
**T1-T2 Change in Social Support (vs. no change)**			
Usual meal consumption^f^			
Meals alone to meals with company	2.98 (0.99–8.94)	1.78 (0.67–4.74)	2.03 (0.30–13.56)
Meals with company to meals alone	2.48 (0.68–9.08)	**4.13 (1.34–12.74)**	**13.78 (1.32–144.36)**
Living situation^f^			
Living alone to living with someone	1.59 (0.12–22.03)	4.83 (1.00–23.30)	0.25 (0.01–5.07)
Living with someone to living alone	0.40 (0.07–2.33)	0.39 (0.08–1.86)	0.97 (0.15–6.35)

OR, Odds ratio; CI, Confidence Interval; MEDAS, Mediterranean Diet Adherence Screener; LTPA, Leisure Time Physical Activity; METS, Metabolic Equivalent of Task, TV, television; PCS, Physical Component Summary; MCS Mental Component Summary; SF-12, the 12-Item Short Form. “—” cell size too small for analysis.

^a^Multivariate ORs are adjusted for all other variables shown in the table, both baseline and change variables, plus alcohol consumption (g/day) at baseline. Statistically significant ORs are shown in **bold**. ^b^“Other” includes other variations in employment status as well as “no change”; ^c^“Lost spouse” due to divorce or death vs. “Other” (got married or no change in status); ^d^The reference category includes participants who reported “no change” and a small number who started smoking; ^e^Change was defined as a decrease or increase of at least 15% of the baseline value; ^f^Baseline variable removed from the model due to collinearity with the change variable; ^g^Adherence to the Mediterranean diet is defined by a MEDAS score ≥9; ^h^Change in BMI was defined as a reduction or increase of at least 5% of the baseline value; ^i^Change was defined as a reduction or increase of number of morbidities by 1 vs baseline. Morbidities included: Pneumonia, asthma, chronic bronchitis, heart attack, stroke, cardiac failure, osteoarthritis, rheumatoid arthritis, hip fracture, gallbladder stones, cirrhosis of the liver, urinary tract infection, depression requiring treatment, Parkinson’s disease, dementia, gum disease, and cancer; ^j^Change was defined as at least a 3-point decline or improvement in score compared to baseline score.

#### Female at risk drinkers

When examining female at risk drinkers at baseline, those 70 and older and those who abandoned the Mediterranean diet were more likely to have decreased their consumption over the 3-year period (OR for At Risk-to-Light: 4.64; 1.51–14.26 and 4.28; 1.06–17.3, respectively). However, those already retired by baseline were more likely to maintain their at risk consumption (OR for At Risk-to-Light: 0.21; 0.05–0.95) than to reduce it.

Finally, as seen above, women classified at risk drinkers who changed meals habits from eating with others at least sometimes to eating always alone, more frequently reduced alcohol intake (OR for At Risk-to-Light: 13.78; 1.32–144.36). However, the large confidence interval points to small cell sizes and, thus, interpretation should be made with caution (Table 7).

## Discussion

In line with previous research supporting increasingly stable alcohol patterns as we age^10^, our results in older adults from Spain show that over a quarter varied consumption patterns during a 3-year follow-up. In contrast, about 50% of Spanish adults under 60 changed drinking patterns over the same period^15^. Also as in previous work^3,6,7,32^, the overall trend denoted a reduction in alcohol intake, except for between one fifth and one fourth of all non-drinkers and 26% of all ex drinkers at baseline who reported measurable alcohol intake 3 years later. This finding is not surprising given the abundant evidence of relapse into alcohol consumption among ex-drinkers, particularly those with a history of problem drinking^33^.

The overall downward trend in alcohol intake (a reduction of 5.5 g/d in average in 3 years) and the gender and educational differences in consumption are consistent with the country profile recently reported by WHO^3^, and several studies in older adults^7,12,20^ but not all^34^. Also it is worth noting that almost a quarter of older adults at follow-up are still drinking substantial amounts, an average of 2 drinks/day (28.5 g/d). In part, it might simply be the continuation of long-held habits^35^ and lack of awareness that consumption deemed safe at younger ages may be harmful later in life, especially if taking certain prescriptions. If that were the case, some Spanish elderly may be amenable to change upon receiving correct information^12^ though reasons for change in consumption levels in older adults vary by age, sex, and social status^32^. Unfortunately, there is also a great deal of skepticism in this population regarding the harmful effects of alcohol^35^.

Our results reveal how changes in consumption patterns vary by baseline characteristics and, further, by concurrent changes in those characteristics throughout the 3 years of the follow-up. For both men and women, indicators of low social connectivity at baseline (living/eating alone) were linked to increasing alcohol consumption to potentially harmful levels, as reported by others^17–19,35,36^ and as supported by the notion that social networks rein in negative or unacceptable health behaviors^37,38^. In contrast, while taking into account loss of spouse/significant other, entering retirement, and changes in perceived mental health-related quality of life, a decrease in social connectivity between baseline and follow-up was accompanied by a reduction in consumption levels. This association challenges the aforementioned studies; however, given we also observed decreased alcohol consumption among men increasing their time watching TV, our findings suggest that substantial reductions in social events (i.e., social connectivity) over a period of time render fewer alcohol consumption opportunities^39^.

Finally, age-associated changes in drinking patterns varied by sex^40^ independently of morbidity and both physical and mental components of health-related quality of life. And, as expected, higher morbidity at baseline and subsequent increases in morbid conditions were associated with reductions in alcohol intake, including quitting consumption, in line with previous studies^41^. Also, the association between reading and healthier drinking patterns in both sexes may reflect higher health literacy levels and/or higher access to health information. The internet has increased the latter greatly; in fact, by 2018 almost half (47%) of Spaniards over 65 had used the internet in the previous 3 months^42^. Regarding health-related changes, changes in BMI and adherence to a Mediterranean diet were related to changes in alcohol intake patterns^43,44^ though the direction of these associations varied by drinking pattern. First, BMI at baseline (independently of BMI changes during the follow-up) was directly and significantly associated with a tendency towards light drinking. Thus, as BMI increased, light drinkers at baseline were more likely to stay light drinkers (vs. quitting) and at risk drinkers were more likely to switch to light drinking by the time of follow-up. In turn, changes in BMI during the study period, independently of baseline BMI, showed paradoxical associations with changes in alcohol patterns. We observed a reduction in BMI for light drinkers who quit alcohol but an increase in BMI for at risk drinkers who reduced consumption to light levels. However, these seemingly contradictory findings mirror the overall inconsistency of this field of enquiry^45^. Previous work shows that the association between alcohol consumption and food intake varies by the individual’s history of alcohol intake. For instance, heavy drinking has been associated with lower energy intake from fat and carbohydrates whereas moderate drinking during meals may increase appetite and caloric consumption^45^. Overall, our results associating lifestyle and health-status variables with alcohol consumption support previously reported clustering of modifiable health risk factors^46^.

Our results should be interpreted in the context of the study’s limitations. First, these analyses are based on a 3-year follow-up. Whereas follow-up periods under 5 years are not uncommon in this field of inquiry^36,38^, it may not have been long enough for assessing the real magnitude of variations in drinking patterns. Also, alcohol consumption was self-reported, which allows for both recall error and social desirability bias. Moreover, our category of non-drinkers may have included a small proportion of sporadic drinkers who, at baseline, reported no consumption due to poor memory recall, and who may have slightly increased consumption (or improved recall) at follow-up just enough to cross over to the category of light drinkers. Further, individuals reporting not consuming alcohol may base their response more on self-comparisons with peers or may consider that daily brandy nightcap as self-medicating (e.g., a sleep-aid) rather than drinking, especially among women^11^. Still, our participants reported not drinking alcohol in very similar proportions as those found in the 2009 European Health Survey in Spain^47^. In this survey 28.5% of men and 64.7% of women aged 65–74 years reported no alcohol consumption in the previous year. In our study, 19.7% of men and 54.1% of women 60 year-old and older were classified as non-drinkers or ex drinkers in the period 2008–2010. Further, Park, Ryu and Cho^30^ followed a community-based cohort of 9,001 Korean men and women for 10 years and reported that 76.2% of abstainers at baseline stayed non-drinkers throughout follow-up, which is very similar to our findings (77.8% for men and 78.0% for women after 3 years). The large percentage of women who did not drink in the past year may have reduced our power in the fully adjusted multivariate analyses to detect actual relationships. Finally, although our analyses included measures of physical health, both objective (number of diagnosed conditions) and subjective (perceived physical quality of life) our results are not adjusted for the number of prescription medications.

Major strengths of this study include a longitudinal design of a representative sample of older adults residing in Spain. This Southern Mediterranean country displays drinking patterns and social drinking contexts which are quite different from those in the U.S. or Northern Europe where most published studies are based on. This rich dataset allowed us to examine associations between changes in social connectivity and drinking patterns in the age-group with the highest proportion of widowhood and “empty-nest syndrome.” At a more methodological level, our measure of average alcohol consumption is based on data from a 12-month detailed validated diet history rather than commonly used 30 day-assessments which are less precise capturing current drinkers and heavy drinkers^48^. Further, our measurement in g/day is an improvement over previous work relying on alcohol data reported in “drinks per day” without specifying the amount of alcohol in one drink [e.g.^12^] or defining one drink as “a glass of wine…a shot of liquor, or a mixed drink with liquor in it” [e.g.^20^] which may vary substantially in glass size and amount of alcohol especially if self-served. Finally, by using the NIAAA classification of safe consumption levels specific for healthy older adults not taking prescribed medications^5^ we were able to identify subtle but clinically significant changes in alcohol intake specific to our population. The use of these lower thresholds rather than those used for younger populations [e.g.^14^] or the NIAAA-endorsed definition of moderate drinking (≤28 g/day and ≤14 g/day of alcohol for men and women, respectively)^49^ was recently supported by strong evidence linking modest consumptions to all-cause mortality data^4^.

In conclusion, whereas prospective research with longer follow-up in this topic is needed, our results indicate that short-term changes in alcohol intake are not infrequent in older ages and that a substantial proportion of older adults are consuming higher amounts of alcohol than recommended. This is especially worrisome given the pervasive underrecognition of alcohol problems^50^ and the high prevalence of polypharmacy (≥5 prescriptions) in adults aged 65 and older both in Spain (21.9%)^51^ as well as in Anglosaxon countries like the United States and England (39.0% and 30.9%, respectively)^52,53^. Ascertaining factors associated with potentially harmful alcohol intake may help health-care providers identify current excessive drinkers or those at risk of becoming one. Finally, these findings may facilitate the development of interventions aimed at minimizing the harmful effects of excessive alcohol intake while maintaining the benefits of socialization, often accompanied by light drinking. Further, strategies should address the various levels of skepticism regarding the harms of alcohol and older people’s susceptibility to “moralizing messaging”^54^. Interventions should take into account that elderly people are a highly heterogeneous population with different life trajectories, distinctive reasons for drinking or not, diverse alcohol consumption histories, and changing circumstances regarding social connectivity. And that how these factors relate to alcohol consumption vary by gender. Thus, customizing prevention messages as to make them relevant to this group’s diverse concerns, specific needs, and daily routine may serve as a starting point in future prevention strategies.

## Data Availability

The data analyzed in this study are available from the corresponding author on reasonable request.
